# Icaritin Inhibits JAK/STAT3 Signaling and Growth of Renal Cell Carcinoma

**DOI:** 10.1371/journal.pone.0081657

**Published:** 2013-12-06

**Authors:** Shasha Li, Saul J. Priceman, Hong Xin, Wang Zhang, Jiehui Deng, Yong Liu, Jiabin Huang, Wenshan Zhu, Mingjie Chen, Wei Hu, Xiaomin Deng, Jian Zhang, Hua Yu, Guangyuan He

**Affiliations:** 1 Genetic Engineering International Cooperation Base of Chinese Ministry of Science and Technology, Key Laboratory of Molecular Biophysics of Chinese Ministry of Education, College of Life Science and Technology, Huazhong University of Science and Technology (HUST), Wuhan, Hubei, China; 2 Department of Cancer Immunotherapeutics and Tumor Immunology, Beckman Research Institute and City of Hope Comprehensive Cancer Center, Duarte, California, United States of America; Children's Hospital Boston & Harvard Medical School, United States of America

## Abstract

Signal transducer and activator of transcription-3 (STAT3) is critical for cancer progression by regulating tumor cell survival, proliferation, and angiogenesis. Herein, we investigated the regulation of STAT3 activation and the therapeutic effects of Icaritin, a prenyl flavonoid derivative from *Epimedium Genus*, in renal cell carcinoma (RCC). Icaritin showed significant anti-tumor activity in the human and mouse RCC cell lines, 786-O and Renca, respectively. Icaritin inhibited both constitutive and IL-6-induced phospho-STAT3 (STAT3^Y705^) and reduced the level of STAT3-regulated proteins Bcl-xL, Mcl-1, Survivin, and CyclinD1 in a dose-dependent manner. Icaritin also inhibited activation of Janus-activated kinase-2 (JAK2), while it showed minimal effects on the activation of other key signaling pathways, including AKT and MAPK. Expression of the constitutively active form of STAT3 blocked Icaritin-induced apoptosis, while siRNA directed against STAT3 potentiated apoptosis. Finally, Icaritin significantly blunted RCC tumor growth *in vivo*, reduced STAT3 activation, and inhibited Bcl-xL and Cyclin E, as well as VEGF expression in tumors, which was associated with reduced tumor angiogenesis. Overall, these results suggest that Icaritin strongly inhibits STAT3 activation and is a potentially effective therapeutic option for the treatment of renal cell carcinoma.

## Introduction

Renal cell carcinoma (RCC) is the most common kidney malignancy and the sixth leading cause of cancer-related deaths [1]. Advanced RCC is highly resistant to conventional therapies, particularly radiotherapy, and thus the utility of other treatments including immune-based therapy have been intensely investigated in clinical trials for metastatic RCC. Although these treatments demonstrate improvements in some patients, complete remission is rarely achieved with IL-2 or IFN-α therapy in advanced RCC, in part due to low overall response rates, systemic toxicities, and resistance [2]. More recent successes have been reported with targeted therapies for RCC, including multikinase inhibitors that block VEGF and mTOR signaling, although responses are short-lived and acquired resistance hampers their overall benefits [3], [4], [5], [6], [7], [8], [9]. Therefore, management of advanced RCC remains a significant clinical challenge and new therapeutic agents that inhibit tumor growth through multiple targeted pathways are urgently needed.

Signal transducers and activators of transcription (STATs) are a family of cytoplasmic proteins that, upon ligand-induced activation by cytokine and growth factor receptors, translocate to the nucleus and regulate transcription of genes involved in diverse cellular activities in diseased states [10], [11], [12]. In particular, signal transducer and activator of transcription-3 (STAT3) is constitutively activated and promotes the development of several solid cancers, including RCC [13,14,15,16,1718]. STAT3 is also reported to have a leading role in cancer inflammation and immunity [19], [20], [21], [22]. Many tumor-derived factors, such as IL-10, IL-6 and VEGF that are crucial for both tumor growth and immunosuppression, activate STAT3 to create an efficient “feed-forward” loop to induce persistent STAT3 activity in tumor cells and the tumor microenvironment [19], [23], [24], [25]. STAT3 is persistently activated by non-receptor tyrosine kinases such as Janus kinases (JAKs) or Src family kinases [13], [19], [26], [27], [28], which induce its dimerization and nuclear translocation required for its transcriptional regulation [13], [19], [29]. The JAK/STAT3 pathway widely reported as a potent pro-survival and pro-metastatic signaling axis, and novel agents that specifically inhibit its activation offer a novel targeted therapeutic approach for many cancers [19], [30], [31], [32].

Icaritin is a hydrolytic product of icarin from *Epimedium Genus*, a traditional Chinese herbal medicine. Icaritin has many pharmacological and biological activities, such as suppression of osteoclast differentiation [33], stimulation of neuronal differentiation [34], [35], and promotion of cardiac differentiation of mouse embryonic stem cells [36]. Icaritin was recently demonstrated to induce apoptosis in human endometrial cancer cells [37], and potently inhibited growth of the breast cancer stem/progenitor cells via inhibition of ERK signaling [38]. In hematological malignancy, Icaritin showed potent anti-leukemia activity in chronic myeloid leukemia *in vitro* and *in vivo* by regulating MAPK, AKT and JAK2/STAT3 signaling pathways [39]. However, the potential therapeutic effects of Icaritin and its key molecular mechanisms have not yet been explored in RCC. Here, we show that Icaritin suppressed both constitutive and inducible STAT3 activation, associated with a reduction in activation of Janus-activated kinase 2 (JAK2). Inhibition of phospho-STAT3 (STAT3^Y705^) by Icaritin reduced the expression of STAT3-regulated cell survival, proliferation, and angiogenic factors. Additionally, Icaritin inhibited tumor angiogenesis, potently suppressed STAT3 activation, and significantly reduced RCC tumor growth *in vivo*. These data suggest that Icaritin is a specific inhibitor of JAK2/STAT3 activation and may represent a viable therapeutic strategy for the treatment of advanced RCC.

## Materials and Methods

### Reagents

Icaritin was purchased from Shanghai Win Herb Medical Science Corporation (China). Anti–phosphorylated Stat3 (p-Stat3; Tyr705), anti–phosphorylated AKT (p-AKT; Ser473), anti-AKT, anti–phosphorylated extracellular signal-regulated kinase1/2 (p-ERK1/2; Thr202/Tyr204), anti-ERK1/2, anti–phosphorylated Janus-activated kinase2 (p-JAK2; Tyr1007/1008) and anti-JAK2 were purchased from Cell Signaling Technology, Inc. Anti-Stat3 (C-20), anti–Bcl-xL (H-5), anti–Cyclin E, anti–Cyclin D1, anti-VEGF, anti–poly(ADP-ribose) polymerase (PARP), human STAT3 small interfering RNA (siRNA) and control siRNA were all purchased from Santa Cruz Biotechnology. Recombinant mouse IL-6 was purchased from Peprotech.

### Cells

Human RCC cell lines 786-O was from ATCC and was grown in RPMI-1640 supplemented with 10% fetal bovine serum (FBS). Cells are serum starved for 24 hours when treated with IL-6. To obtain the STAT3C-expressing cells, 786-O cells were transiently transfected with plasmids containing pRC/CMV-vector and pRC/CMV-STAT3C-Flag using Lipofectamine 2000 according to the manufacturer's protocol (Invitrogen). The murine cell line Renca was also obtained from ATCC and was grown in RPMI 1640 supplemented with 10% FBS.

### Proliferation assay

Cell proliferation was measured with Cell Titer 96 Aqueous One Solution Cell Proliferation Assay (Promega), which contains MTS. 96-well plates were seeded with 3,000 cells per well in RPMI-1640 supplemented with 1% FBS. After overnight incubation, cells were treated with varying concentrations of Icaritin (1∼10 µM) or DMSO control. After 24- or 48-hours, MTS was added to the cells according to the manufacturer's protocol and absorbance was measured at 490 nm using an automated ELISA plate reader (Molecular Devices).

### Apoptosis assay

786-O or Renca cells (2×10^5^) were seeded in 60-mm culture dishes in RPMI-1640 with 1% FBS. The following day, cells were treated with indicated concentrations of Icaritin for 24-hours. After treatment, floating and attached cells were collected and stained with PI and Annexin V-FITC Apoptosis Detection kit (BD Biosciences) in FACS Wash Buffer (HBSS^−/−^ containing 2% FBS) according to the manufacturer's instruction. Viable and apoptotic cells were analyzed by flow cytometry (Accuri C6). Data was analyzed using FlowJo software (Treestar).

### Western blot

Total protein (20 µg) was resolved by sodium dodecyl sulfate–polyacrylamide gel electrophoresis and transferred to a polyvinyllidene difluoride membrane. Membranes were blocked for 1 hour at ambient room temperature (ART) in 10% non-fat dry milk in TBST (1×TBS with 0.1% Tween 20) followed by an overnight incubation at 4°C with primary antibodies in TBST with 5% BSA. Horseradish peroxidase–labeled anti-mouse or anti-rabbit secondary antibodies were added for 1 hour at ART and detected with Super Signal West Pico substrate (Pierce). Bands were measured as optical density using ImageJ software. The optical density of each band was normalized by β-actin optical density.

### Plasmid transfection

786-O cells were transiently transfected with human STAT3 siRNA and control siRNA using Lipofectamine™ 2000 (Invitrogen). After 24 hours transfection, cells were treated with Icaritin or DMSO control for 24 hours and cell viability was measured.

### *In vivo* experiments

Female BALB/c mice (7–8 week old) were purchased from NCI. Animal use procedures were approved by the institutional committee of the Beckman Research Institute at City of Hope Medical Center. Mice were implanted s.c. with 2.5×10^6^ Renca cells. After tumors reached 5 to 7 mm in diameter, Icaritin or vehicle (DMSO) control was administered peritumorally once every other day at 10 mg/kg body weight. Tumor growth was monitored every other day with digital caliper measurements.

### Immunofluorescence staining

Frozen sections of vehicle control- and Icaritin-treated tumors were stained for CD31/PECAM-1 (BD Biosciences) and Hoechst 33342, and images were acquired using the Zeiss LSM510 upright confocal microscope. Images were analyzed using ImagePro and ImageJ software.

### Statistical analysis

Data are represented as mean ± SD or SEM where indicated, and statistical comparisons were performed using Student's *t*-test for determination of p-values.

## Results

### Icaritin inhibits proliferation and induces apoptosis in RCC cells

**Figure 1:**
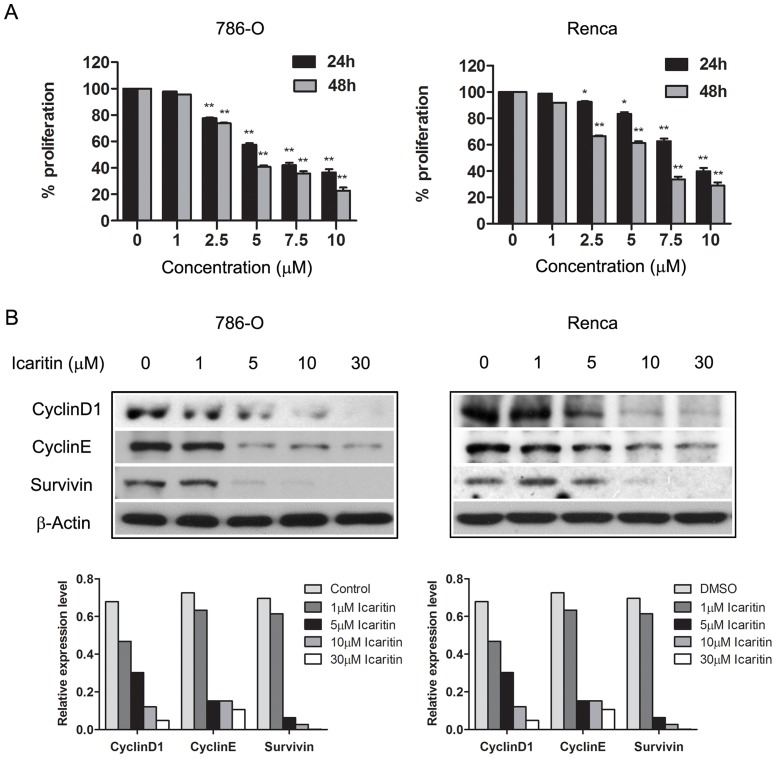
Icaritin inhibits 786-O, Renca cell proliferation. A. Analysis of RCC cell proliferation following treatment of Icaritin. Renca and 786-O cells were treated (24 and 48 hours) at increasing doses (1∼10 µM). Cell proliferation was evaluated by MTS assay. Columns, mean (n = 3, in triplicate); bars, SD. *, P<0.05; **, P<0.01. B. Icaritin treatment of 786-O cells reduces expression of proliferative proteins. Western blot analyses of 786-O cells treated (24 hours) with Icaritin to evaluate protein levels of cyclinD1, cyclinE and survivin. Bottom. The optical density of each protein was quantified by β-actin optical density.

To determine whether Icaritin has direct anti-tumor effects in RCC cells, dose-response and time course studies were performed in human 786-O cells and in mouse Renca cells. Cells treated with Icaritin showed significant inhibition of cell proliferation in a dose- and time-dependent manner, blocking proliferation over 60% with 10 µM (Fig. 1A). Western blotting was also performed to determine the downstream factors mediating the effects of Icaritin on RCC cells. The results showed that Icaritin treatment of 786-O and Renca cells reduced expression of several key anti-apoptotic and pro-proliferative proteins, including cyclin E, cyclin D1, and survivin (Fig. 1B).

**Figure 2:**
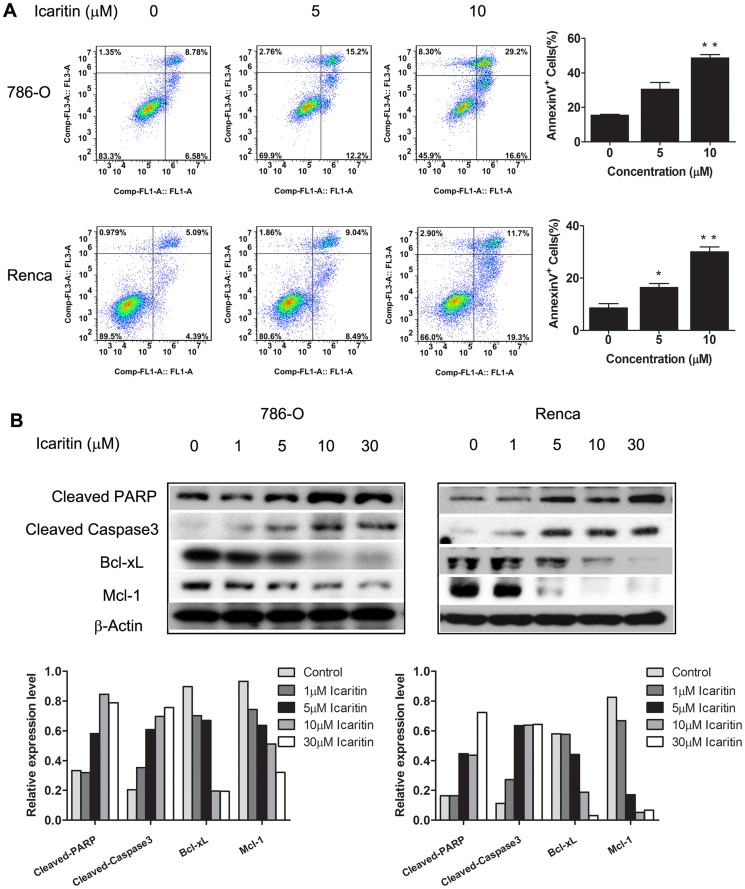
Icaritin induces apoptosis in 786-O, Renca cells. A. Analysis of RCC cell apoptosis following treatment of Icaritin. Renca and 786-O cells were treated (24 hours) at indicated doses, harvested, and stained with Annexin V-FITC and PI. Annexin V-FITC positive apoptotic cells were determined by flow cytometry. Columns, mean (n = 3, in triplicate); bars, SD. B. Icaritin treatment of RCC cells regulate the expression of apoptosis related proteins. Western blot analyses of 786-O cells treated (24 hours) with Icaritin, to evaluate protein levels of total and cleaved PARP, cleaved caspase3, Bcl-xL, and Mcl-1. Bottom. The optical density of each protein was quantified by β-actin optical density.

We next investigated whether Icaritin induced apoptosis in RCC cells. After treatment with Icaritin for 24 hours, 30% and 50% of 786-O and Renca tumor cells, respectively, were Annexin-V positive as defined by flow cytometry (Fig. 2A). To confirm apoptosis in Icaritin-treated RCC cells, we detected the levels of activated caspase-3 and PARP cleavage. Icaritin increased cleaved caspase-3 and cleaved PARP, along with decreased Bcl-xL and Mcl-1, in a dose-dependent manner (Fig. 2B). Collectively, these data indicate that Icaritin has potent anti-tumor and pro-apoptotic effects on human and mouse RCC cells.

### Icaritin inhibits JAK2/STAT3 signaling in RCC cells

**Figure 3:**
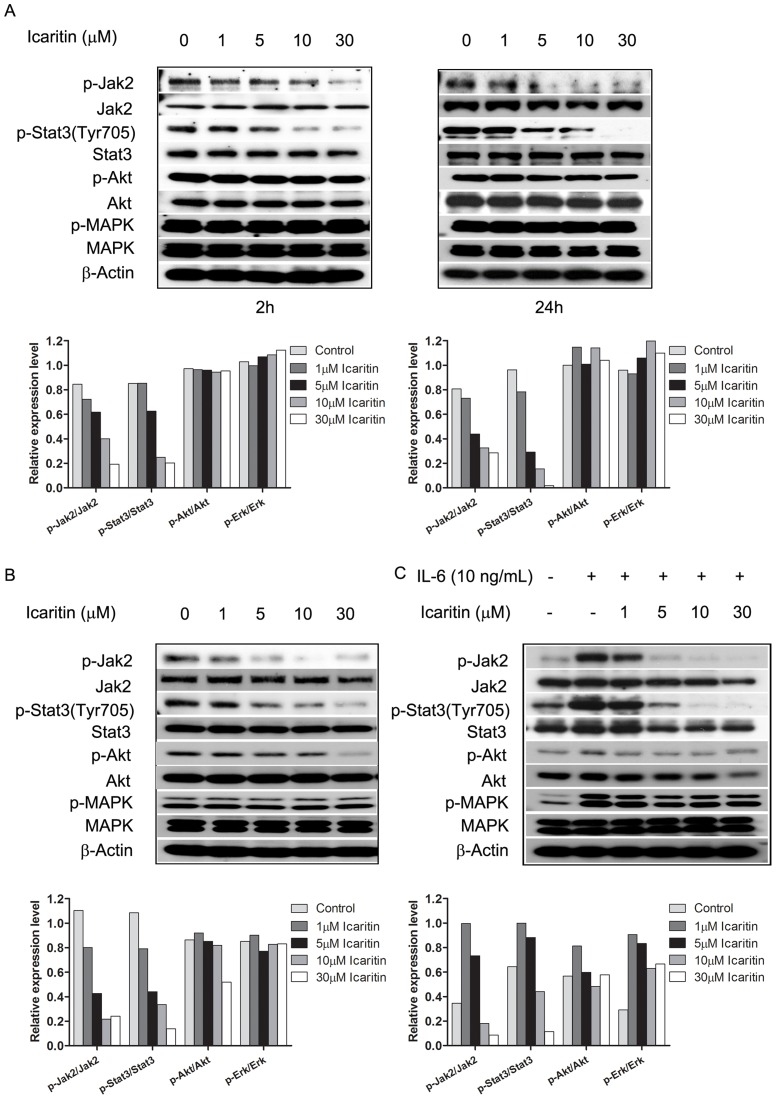
Effects of Icaritin on major oncogenic signaling pathways in 786-O and Renca cells. Icaritin reduced STAT3 and JAK activity, with no dramatic reduction of AKT, MAPK signaling in 786-O (A) and Renca (B, C) tumor cells. Tumor cells were treated with Icaritin at indicated concentrations for 2 or 24 hours. Total cell lysates were prepared and Western blots were performed using relevant antibodies to detect total protein levels, with β-actin used as the loading control. C. Pre-incubation of Icaritin inhibited the phosphorylation of STAT3 (Tyr705) induced by IL-6. To assess whether icaritin inhibits the phosphorylation of STAT3 at Tyr705 induced by IL-6, Renca cells were serum-free starved for 24 hours, and treated with Icaritin for 2 hours followed by the addition of IL-6 (10 ng/mL) for 20 minutes. Anti–β-actin monoclonal antibody was used as a loading control. Bottom. The optical density of each protein was quantified by β-actin optical density.

To explore the underlying mechanisms regulating the effects of Icaritin on RCC cells, we examined several major oncogenic signaling pathways, including STAT3, AKT, and mitogen-activated protein kinase (MAPK) [14], [40], [41]. Given that STAT3 is constitutively activated in diverse cancers, including RCC, we assessed whether Icaritin-induced tumor cell death was associated with STAT3 inhibition. Although Icaritin had no effects on total STAT3 protein levels in tumor cells, it inhibited activated STAT3 as early as 2 hours after Icaritin treatment, with continued inhibition of STAT3 activation after 24 hours (Fig. 3A). The early inhibition of STAT3 activity correlated well with Icaritin-induced inhibition of tumor cell proliferation.

We further assessed the potential effects of Icaritin on the status of JAK2, which is also frequently activated in cancer cells. 786-O tumor cells contained constitutively activated p-JAK2, which was dose-dependently inhibited by Icaritin (Fig. 3A). Similar results were obtained in Renca cells (Fig. 3B). Of note, there were minimal effects on p-AKT and p-ERK1/2 levels in 786-O cells following 2-hour Icaritin treatment (Fig. 3A). These data demonstrate a specific inhibitory effect of Icaritin on JAK2/STAT3 activation in RCC cells. Because IL-6 is a potent growth factor for RCC cells and its effect are primarily mediated through activation of STAT3 [42], [43], we determined whether Icaritin could inhibit IL-6–induced STAT3 phosphorylation. Renca cells pretreated with Icaritin for 2 hours and then stimulated with IL-6 (10 ng/mL) for 20 minutes demonstrated a significant reduction in JAK2/STAT3 signaling compared with IL-6 stimulation alone (Fig. 3C). The results suggest that Icaritin inhibits constitutive JAK2/STAT3 activation, as well as IL-6-induced JAK2/STAT3 (Fig. 3C). Interestingly, Icaritin also slightly inhibited IL-6-induced p-AKT and p-MAPK. It may due to the crosstalk of different signal pathways.

### Icaritin-induced apoptosis is regulated by STAT3 signaling in RCC cells

**Figure 4:**
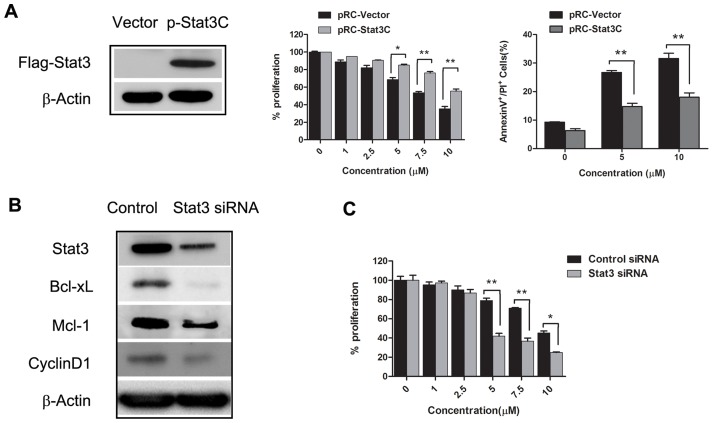
Levels of Stat3 activity affect the direct antitumor effects of Icaritin. A. Over expression of a constitutively activated STAT3 (STAT3C) rescues 786-O cells from apoptosis induced by Icaritin. Pooled 786-O tumor cells containing a control vector, pRC-vector, or the pRC-STAT3C expression vector were treated (24 hours) with Icaritin at different concentrations (0, 1, 5, 10, 30 µM). Left. The success of transfection was confirmed by immunoblotting assay with FLAG antibody. Middle. Cell proliferation was analyzed by MTS assay. Right.Tumor cells positive for both Annexin V and PI, as determined by flow cytometry, were considered apoptotic. Columns, mean (n = 3, in triplicate); bars, SD. *, P<0.05; **, P<0.01. B. STAT3 inhibition reduces expression of genes important for proliferation. 786-O tumor cells were transfected with STAT3 or control siRNAs and total cell lysates were collected 24 hours after transfection. Western blot analyses of lysates with indicated antibodies. C. Knockdown of STAT3 enhances the effects of Icaritin on 786-O tumor cell growth arrest. 786-O tumor cells transfected with either control or Stat3 siRNA followed by treatment (24 hours) with Icaritin at indicated doses. Cell proliferation was analyzed by MTS assay. Columns, mean (n = 3, in triplicate); bars, SD. *, P<0.05; **, P<0.01.

To further investigate whether STAT3 activity directly influences the biological effects of Icaritin in RCC cells, an expression vector encoding a constitutively-active STAT3 mutant, STAT3C [44] or an empty control vector (pRC) were transfected into RCC cells. Transfected cells were confirmed by Western blot analysis (Fig. 4A Left). Expression of constitutively-active STAT3 in 786-O cells promoted resistance to the anti-proliferative and pro-apoptotic effects of Icaritin (Fig. 4A Right). Our initial results (Fig. 1B) showed that Icaritin treatment inhibited several STAT3-regulated proteins important for tumor cell survival and proliferation. In agreement with this finding, siRNA-mediated knockdown of STAT3 in 786-O cells significantly reduced the expression of several known STAT3 downstream genes, including Mcl-1, cyclinD1 and Bcl-xL (Fig. 4B). We further demonstrated that siRNA-mediated knockdown of STAT3 sensitized RCC cells to the anti-proliferative effects of Icaritin (Fig. 4C).

### Icaritin inhibits tumor growth and angiogenesis *in vivo*

**Figure 5:**
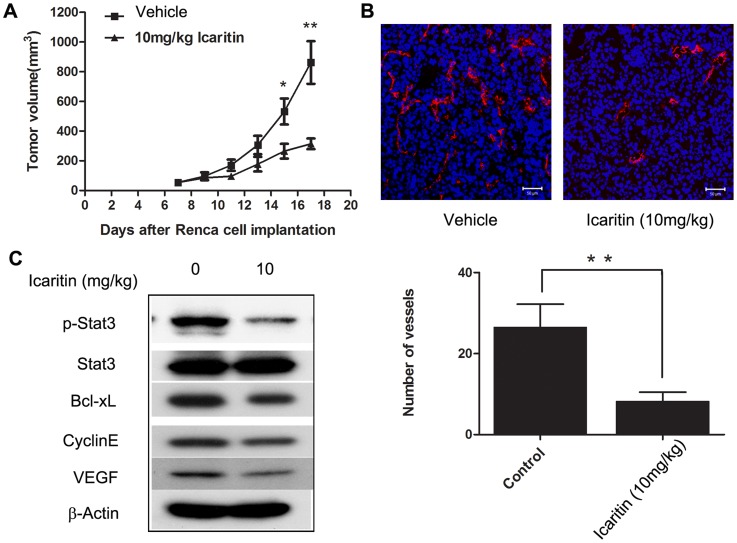
Icaritin inhibits Renca tumor growth and vessels, which corresponds to p-Stat3 and VEGF reduction. A. Icaritin inhibits Renca tumor growth. BALB/c mice were implanted s.c. with Renca cells (2.5×10^6^). Icaritin or vehicle control was administered peri-tumor every other day at the indicated doses 7 days after tumor challenge. Points, mean (n = 6); bars, SE. P<0.01. B. Icaritin inhibits p-STAT3 protein level and reduces Bcl-xL, cyclinE and VEGF expression in Renca tumors. Western blot analyses of tumor tissues harvested 10 days after Icaritin treatment using indicated antibodies. C. Top. Frozen tumor sections of vehicle- and Icaritin-treated tumors (same as in A) were stained for CD31/PECAM-1 (red) and Hoechst 33342 (blue) and analyzed by confocal laser scanning microscopy. Scale bar, 50 µM. Bottom. Number of vessels in at least five sections (10× magnifications) per tumor was used for quantification. Columns, mean (n = 4); bars, SD. **, P<0.01.

We next assessed whether Icaritin inhibits tumor growth *in vivo* using immunocompetent mice bearing Renca tumors. Icaritin treatment (10 mg/kg) of Renca tumor-bearing mice resulted in potent inhibition of tumor growth (Fig. 5A), which correlated with a reduction in STAT3 activity in tumors (Fig. 5B) and a reduction in Bcl-xL and Cyclin E protein expression (Fig. 5B). Moreover, body weight loss was not observed in mice treated with Icaritin. At the end of the experiment, in the Icaritin groups, the body weight was 21.3±0.25 g, which is comparable to the control group 21.5±0.49 g. There was no statistical difference between Icaritin-treated and control group.

Additionally, VEGF expression was significantly reduced in tumors of mice treated with Icaritin, indicating a potential effect of Icaritin on tumor angiogenesis. To further investigate the anti-angiogenic effects of Icaritin, we assessed blood vasculature in tumors of mice treated with Icaritin. As shown in Figure 5C, we demonstrated a significant reduction in CD31^+^ vessels in tumors treated with Icaritin compared with vehicle control. Taken together, these data indicate that Icaritin inhibited tumor STAT3 activity, resulting in significantly reduced tumor growth and inhibition of tumor vasculature in RCC tumors *in vivo*.

## Discussion

Activated STAT3 promotes tumorigenesis by preventing apoptosis and enhancing proliferation, angiogenesis, invasiveness, and immune evasion [21], [45], [46], [47], [48]. In various cancer types, including leukemias and solid cancers of the breast, head and neck, melanoma, prostate, pancreas, and colon, aberrant activation of STAT3 crucially contributes to cancer progression [13]. STAT3 is constitutively activated in human RCC and is an independent prognostic indicator [14], [49], [50], [51]. It has been reported that STAT3 is a potential modulator of HIF-1-mediated VEGF expression in human renal carcinoma cells [52], suggesting that STAT3 represents a promising therapeutic target for the treatment of RCC. Several small molecule inhibitors induce apoptosis and have been associated with inhibition of STAT3 activation in RCC [15], [53]. In particular, it was recently reported that WP1066, a STAT3 inhibitor, reduced RCC tumor growth and metastasis [15], but whether this inhibitor required STAT3 for its anti-tumor effects was not directly assessed. Icaritin, a novel natural herbal product derivative, has been recently reported with anticancer effects, inhibiting growth of breast cancer, endometrial cancer and chronic myeloid leukemia cells [37], [38], [39]. To our knowledge, this is the first report demonstrating therapeutic effects of Icaritin on RCC. Our results demonstrate that Icaritin inhibits STAT3 activation, in part through inactivation of upstream JAK2 in RCC cell lines, 786-O and Renca.

In cancer cell lines and in patient tumor tissues, there is evidence for constitutive activation of STAT3 through chronic cytokine stimulation through autocrine and/or paracrine loops, often involving IL-6 [13], [19], [21]. IL-6 binds to the sIL-6R receptor (gp80, present either as a soluble or cell-surface protein), which then induces dimerization of gp130 chains resulting in activation of the associated Janus kinases (JAKs). JAKs phosphorylate gp130, leading to the recruitment and activation of the STAT3, which then leads to STAT3-mediated transcriptional regulation [13], [19], [21], [54]. In our study, we found that Icaritin dramatically inhibited IL-6-induced STAT3 activity, associated with upstream JAK2 inhibition. Icaritin also modestly inhibited IL-6-induced p-AKT and p-MAPK, which may be attributed to crosstalk of different signal pathways under stimulated conditions. Although further studies are required to determine the exact mechanisms of action, Icaritin potently inhibits the JAK2/STAT3 signaling axis to block IL-6-induced protein expression.

We further demonstrated that the anti-proliferative and pro-apoptotic effect of Icaritin in RCC cells was mediated, in part, by inhibition of STAT3 activation. Activated STAT3 has been shown to protect tumor cells from apoptosis by inducing proliferation/survival genes and blunting pro-apoptotic genes [13], [45]. Several of these key signaling factors, including Cyclin D1, Bcl-xL, and Mcl-1, were also reduced in a dose-dependent manner by Icaritin. Confirming our *in vitro* findings, we show significant inhibition of tumor growth and angiogenesis by Icaritin in a mouse model of RCC.

Metastatic RCC is highly refractory to conventional radiation therapy and chemotherapy [55]. Recent successes have been reported with targeted therapies for RCC, but the responses are short-lived and acquired resistance hampers their overall benefits [3]–[9]. The management of advanced RCC therefore remains a significant clinical challenge. Because of their safety and ability to affect multiple targets, natural products will likely continue to be intensely investigated for use in the treatment of various cancers, including metastatic RCC. Our study highlights Icaritin as a natural product in treating metastatic RCC through inhibition of JAK/STAT3 signaling.
